# Laser Therapy for the Treatment of Morphea: A Systematic Review of Literature

**DOI:** 10.3390/jcm10153409

**Published:** 2021-07-30

**Authors:** Paulina Szczepanik-Kułak, Małgorzata Michalska-Jakubus, Dorota Krasowska

**Affiliations:** Chair and Department of Dermatology, Venerology and Paediatric Dermatology, Medical University of Lublin, 20-081 Lublin, Poland; mjm@poczta.onet.eu (M.M.-J.); dor.krasowska@gmail.com (D.K.)

**Keywords:** morphea, localized scleroderma, laser therapy

## Abstract

Morphea, also known as localized scleroderma (LoS), comprises a set of autoimmune sclerotic skin diseases. It is characterized by inflammation and limited thickening and induration of the skin; however, in some cases, deeper tissues might also be involved. Although morphea is not considered a life-threatening disease, the apparent cosmetic disfigurement, functional or psychosocial impairment affects multiple fields of patients’ quality of life. Therapy for LoS is often unsatisfactory with numerous treatments that have only limited effectiveness or considerable side effects. Due to the advances in the application of lasers and their possible beneficial effects, the aim of this study is to review the reported usage of laser in morphea. We present a systematic review of available literature, performed with MEDLINE, Cinahl, Central, Scopus, Web of Science, and Google Scholar databases. We identified a total of twenty relevant studies (MEDLINE *n* = 10, Cinahl *n* = 1, Central *n* = 0, Scopus *n* = 2, Web of Science *n* = 5, Google Scholar *n* = 2) using laser therapy for LoS. Eight studies were focused on the use of PDL, six on fractional lasers (CO_2_ and Er:YAG), four on excimer, and two on either alexandrite or Nd:YAG.

## 1. Introduction

Morphea, also known as localized scleroderma (LoS), encompasses a spectrum of autoimmune cutaneous connective tissue diseases. It is characterized by inflammation, excessive collagen production, and subsequently limited sclerosis of the skin; however, in some cases, deeper tissues such as subcutaneous fat, fascia, muscles, or bones might also be involved. The incidence of morphea is estimated at 27 cases/1,000,000, with greater predominance among females (women are 2.6–6.0 times more frequently affected than men) [1]. According to the German guideline that considers the degree of fibrosis, five types of LoS are distinguished: limited, generalized, linear, deep, and mixed, with the linear subtype predominating in children [2], and circumscribed subtype more common in adults [3]. The etiopathogenesis of LoS has not been fully understood. It is hypothesized to arise as a result of interactions between genetic, epigenetic, environmental, and immunological factors [4].

An active phase of morphea represents limited inflammation, presenting as red or violet patches followed by characteristic porcelain-white or wax-yellow sclerotic plaques, often accompanied by subjective symptoms such as pruritus and/or pain. After several months or years, sclerotic plaques resolve, but skin and/or deeper tissues atrophy and dyspigmentation remain as an atrophic stage. Some severe types, particularly generalized and linear, may be associated with various extracutaneous manifestations [5,6]. It is worth emphasizing that histopathological features mirror the disease phase, although a diagnostic skin biopsy should be taken only in case of an unclear clinical presentation. Early, active lesions present with thickened and homogenized bundles of collagen as well as perivascular infiltrate mostly combined of lymphocytes and plasma cells, alternatively eosinophils and monocytes. The epidermis is usually atrophic. As the disease progresses, homogenization and hardening of collagen appear, which leads to rarefaction of the dermal blood vessels and eccrine glands. The differential diagnosis of LoS should include specific staining: orcein, trichrome, alcian blue, or immunohistochemical for CD34 and factor XIII [5,6].

Clinical evaluation of skin involvement in morphea should be carried out with the use of the ‘localized scleroderma cutaneous assessment tool’ (LoSCAT), that is a standard tool, together with general medical evaluation (Physician’s Global Assessment, PGA) [7]. LoSCAT is a combination of the ‘modified localized scleroderma severity index’ (mLoSSI) and ‘localized scleroderma skin damage index’ (LoSDI). mLoSSI assesses signs of disease severity/activity, including erythema, skin induration, and development of new lesions or expanding pre-existing ones during the last month, whereas LoSDI encompasses dermal and subcutaneous atrophy and pigment changes (hyper-/hypopigmentation) [8].

Appropriate treatment of LoS should be preceded by determining the disease type, activity, severity, and extent of lesions. In mild, limited, and superficial skin lesions, topical treatments (glucocorticoids, calcipotriol, calcineurin inhibitor), or UV phototherapy (UVA1 or PUVA) are sufficient, whereas widespread disease, linear, or deep types require systemic treatment (methotrexate, glucocorticoids). Surgical procedures are primarily dedicated to patients with linear types of LoS, but only in the inactive stage of the disease [3]. 

Although morphea is not considered a life-threatening disease, the apparent cosmetic disfigurement and functional or psychosocial impairment affect the patients’ quality of life [9]. In this field, medical treatments have often proved unsatisfactory and do not meet patients’ expectations of cosmetic outcome. Thus, it seems obvious that looking for safe and effective practices that correct aesthetic and functional deficits is highly desirable [10,11]. Due to advances in the application of lasers and their possible beneficial effects, the aim of this study is to review the reported usage of lasers in treating morphea.

## 2. Materials and Methods

The systematic review of the literature was performed with MEDLINE, Cinahl, Central, Scopus, Web of Science, and Google Scholar databases complementary to PRISMA (Preferred Reporting Items of Systematic Reviews and Meta-Analyses) protocol. The inclusion criteria for considering studies for the review based on PICOS structure comprised the population of pediatric and adult patients diagnosed with morphea and undergoing laser treatment. The search was performed in July 2021 without a date limit. The databases were searched using the relevant MeSH terms: “morphea” OR “localized scleroderma” AND “laser”. The last day to access the databases was 15 July 2021.

## 3. Results

**Figure 1:**
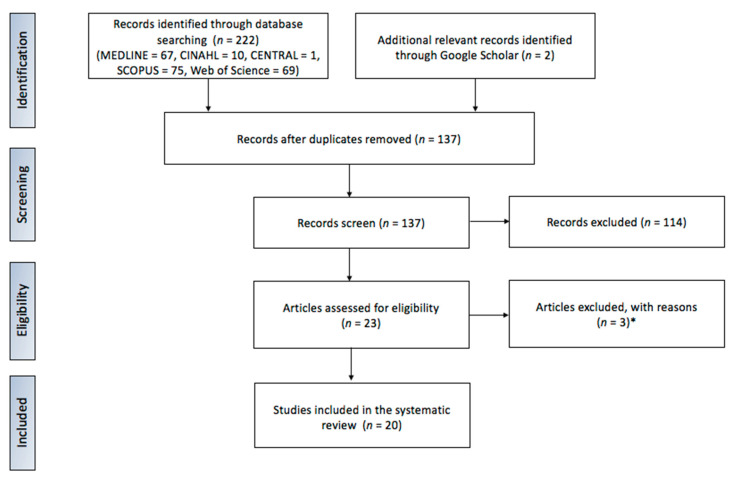
Literature search based on PRISMA protocol. * Articles were excluded due to their purely review nature.

The initial search revealed 222 papers. All of them were checked for the relevance of the main topic. Ultimately, twenty papers (MEDLINE *n* = 10, Cinahl *n* = 1, Central *n* = 0, Scopus *n* = 2, Web of Science *n* = 5, Google Scholar *n* = 2) published between 2000 and 2021 were taken for the final analysis (Figure 1).

The number of reported patients ranged from 1 (case reports) up to 26. 

### 3.1. Types of Lasers Used for LoS Treatment

#### 3.1.1. Pulsed Dye Laser (PDL)

PDL was implemented in the 1980s. Its mode of action is based on the principle of selective photothermolysis, a process of selective damage to chromophores by monochromatic light. PDL targets oxyhemoglobin, resulting in coagulation, endothelial cell injury, and some photochemical effects. The most frequently used wavelengths are 585 and 595 nm, penetrating up to 1.5 mm deep into the skin [12,13]. It is used primarily for the treatment of cutaneous vascular lesions, such as port-wine stains (PWS), hemangiomas, spider angiomas, and telangiectasias. Other uses of PDL in clinical dermatology include nonvascular and inflammatory disorders, in particular psoriasis, acne, chronic discoid or systemic lupus erythematosus, lupus tumidus, granuloma faciale, cutaneous sarcoidosis [13], ecchymoses, striae, warts, contagious molluscum, and basal cell carcinoma [12]. Importantly, several studies have previously shown an improvement of texture and pliability of hypertrophic scars, keloids, and other fibrotic skin lesions with PDL therapy. [14,15,16]. The mechanism of action of 585 nm PDL in LoS is uncertain. Following the principle of selective photothermolysis and vascular selectivity, the microvascular thermal damage may affect either collagen or matrix metalloproteinases’ (MMPs) activity that allows its remodeling [17,18,19].

Based on a literature search, we found eight studies on PDL in morphea. Eisen et al. were the first to report the use of 585 nm PDL in LoS in 2002 [20]. They described a 41-year-old woman with biopsy-proven 3 cm plaque-type morphea (characterized with atrophy, fibrosis, and hyperpigmentation) treated with four sessions of PDL bimonthly. Treatment resulted in improvement in skin pigmentation and noticeable softening of the morphea lesion after each session, which was sustained after a 6-month follow-up, without any adverse effects [20]. Subsequently, Tawfik et al. evaluated the therapeutic effectiveness of PDL in a series of 26 patients with LoS, which failed to improve with other treatment modalities, and compared with 10 healthy subjects [21]. In contrast to Eisen et al., authors performed PDL therapy fortnightly until the obvious softening of induration was achieved (ranging from 4 to 12 sessions). At the end of the treatment, 50% of patients showed complete resolution of induration, while 27% had only a mild degree of improvement. Importantly, 23% of patients with most severe induration at baseline (73% of included cases), experienced a statistically significant although moderate improvement that persisted during the follow-up at 3, 6, and 12 months. The advantage of the study was that clinical assessment was combined with histological and immunohistochemical analyses of lesional skin. Clinical improvement was reflected by histologic changes, particularly a statistically significant decrease in collagen density with the reconstruction of skin adnexa. Authors suggested that the heat produced by PDL led collagen bundles to shrink. Additionally, the number of blood vessels in the upper and lower dermis increased noticeably. Furthermore, immunohistochemical observation showed an increase in the number of dendritic CD34^+^ cells in either the upper or lower dermis, but only the latter was statistically significant. These cells in the course of LoS are involved in fibroblast activation. Their decline indicates the diagnosis of LoS, and allows assessment of the effectiveness of applied therapy [21]. Based on this study, a direct correlation between duration of the disease, as well as the size of the lesion, and the collagen improvement rate after laser therapy may be concluded. It should also be emphasized that the treatment was well tolerated by the patients, as 22 of them reported satisfaction with the results [21].

However, some limitations should be highlighted. A few cases have been reported of inflammatory morphea initially treated with PDL. Kakimoto et al. [22], Kim et al. [23], Pickert et al. [24], Nijhawan et al. [25], Ng et al. [26], and Miura et al. [27] published six single-case reports of females aged between 2.5 to 24 years, with early inflammatory morphea (of durations ranging from 3 months to 2 years) presenting as erythematous patches that were misdiagnosed as vascular lesions, namely PWS. Laser treatment was started based only on the clinical picture, before the confirmative biopsy results or the appearance of characteristic features of LoS (induration, atrophy). The treatment was partially effective with various degrees of reduction in skin erythema (lesions were noted to lighten), but the alleviation was temporary and did not prevent subsequent induration. In addition, Kakimoto et al. observed an exaggerated response to PDL treatment with focal erosions and crusting that, in the authors’ opinion, might be a clinical clue to differentiate early localized scleroderma and true vascular lesion [22]. Of note, early LoS lesions are likely to mimic vascular lesions, so when LoS is suspected, it is pivotal to undertake a thorough differential diagnosis to avoid any therapeutic missteps.

#### 3.1.2. Excimer Laser

The excimer laser uses a mix of reactive and inert gases. When electrically excited, the gas mixture emits a monochromatic, coherent wavelength laser beam (308 nm), making very precise, minute changes to irradiated material. Keratinocytes and T-cells absorb the light, promoting DNA damage and reducing local inflammation and keratinocyte activity [28]. Given the many advantages of an excimer laser, such as a limited course of treatment, lower ultraviolet dose exposure, and direct way of acting, this has led to their widespread use in many focal skin diseases characterized by inflammation or hypopigmentation (psoriasis, vitiligo, atopic dermatitis, alopecia areata, or cutaneous T-cell lymphoma) [29,30].

The excimer laser was also successfully used to treat morphea lesions. In 2009, Nisticò et al., in a study of excimer light in skin diseases, observed that three out of five patients with LoS experienced partial clinical remission with marked improvement in skin texture after 8–12 treatment sessions; however, it was accompanied by residual hyperpigmentation [31]. Later, Hanson et al. presented a pediatric patient receiving excimer laser therapy biweekly and systemic methotrexate for a total of 7 months due to linear scleroderma, unresponsive to the previous treatments (calcipotriene ointment, intralesional triamcinolone acetonide). On such combined therapy, lesions became inactive anddecreased in size, with alleviated subjective symptoms, but unfortunately, comparable results for laser monotherapy are not available from this study [32]. Similarly, Hajjar et al. used an excimer laser twice a week in a 28-year-old woman with active morphea lesions located on the left side of the neck, who was previously unsuccessfully treated with topical steroids and methotrexate [33]. In this case laser therapy was introduced as a complementary treatment to less effective hydroxychloroquine and calcipotriene/betamethasone ointment. As a result, after two months of 16 treatment sessions, peripheral inflammatory erythema (“lilac ring”) resolved, and no progression of current lesions was noted [33].

The efficacy of excimer lasers in morphea mainly results from their anti-inflammatory action, since the 308 nm wavelength used in the excimer laser is the most efficient wavelength for inducing DNA breakage in lymphocytes [34]. Thus, although sparse clinical data, the excimer laser treatment seems to represent a valuable management option for active lesions refractory to previous therapies or as an adjunct to conventional treatment, potentiating the therapeutic effect. In fact, Tatu et al. in their recent review, recommended 308 nm excimer laser therapy as an effective and safe treatment of superficial inflammatory LoS lesions, i.e., erythematous plaques or oedematous patches, especially when the topical corticosteroids or UVA-therapy do not give clinical improvement [34]. Basically, if therapy with UVB or narrow-band UVB is recommended, the excimer laser represents a valuable therapeutic alternative [34].

#### 3.1.3. Carbon Dioxide (CO_2_) or Erbium-Doped Yttrium Aluminum Garnet (Er:YAG) Fractional Lasers

The mode of action of fractional lasers is based on fractional photothermolysis, which means generating in the skin a mesh of microthermal treatment zones surrounded by healthy, undamaged tissue [35,36]. This promotes proper wound healing, which is of particular interest in the case of morphea patients who are at risk of impaired regeneration of traumatic skin lesions [37,38]. Histologically, the process of reepithelization starts within one or two days after the laser treatment. During the first week, epidermal cells invaginate into the microthermal treatment zones. Afterward, these zones are replaced with freshly synthesized collagen, further undergoing long-term remodeling for 6 months or even longer after a session [39]. This process is coordinated by heat shock proteins, matrix metalloproteinases, growth factors, and other mediators [40,41,42,43,44,45]. Fractional lasers have been reported effective in reducing symptoms of acne lesions, stretch marks, melasma, lichen planus, lichen sclerosus, or sclerodermatous chronic graft versus host disease [46,47]. Of note, the successful use of ablative fractional lasers in the treatment of traumatic scar contractures has been described, indicating that such therapy might also be effective in other fibrotic conditions such as morphea [48,49,50]. In this aspect, fractional lasers may also be used to facilitate transdermal and intradermal drug delivery (laser-assisted drug delivery) to the dermis [38].

In the available literature, there are reports on 24 cases of patients with LoS who have been successfully treated with fractional lasers. For the first time Kineston et al. presented a case of successful use of fractional ablative 10.6-µm carbon dioxide (CO_2_) laser in a 27-year-old woman with linear bandlike sclerotic plaque extending from the medial thigh across the knee and ankle joint to the mediodorsal foot, complicated by a left foot contracture (plantar flexion limited to 45°) which failed to improve after several months of combination therapy with UVA-1, topical calcipotriene, 0.005%, cream, and methotrexate 20 mg/week [51]. The addition of a single CO_2_ laser session resulted in an immediate subjective improvement in the range of foot motion, a 5° increase in plantar flexion as soon as after 1 week, and remarkable softening of the sclerotic band with the recovery of full plantar flexion of the involved foot at 4 months after the laser treatment, that was maintained after a 1-year follow-up. The treatment was well tolerated by the patient [51]. Farmer et al. reported a case series of two patients with linear morphea of the head or thigh, who were treated with one or five sessions of deep fractionated CO_2_ laser, respectively [52]. In the case of a head lesion, laser therapy was followed by 0.3 cc topical poly-L-lactic acid and botulinum toxin A. It is noteworthy that, in both cases, discoloration, asymmetry, induration, and mobility significantly improved, thus indicating the potential effect of laser therapy independent of site-involved or adjuvant treatments [52]. Similarly, Yeager et al. reported successful treatment of deep morphea on the shoulder with fractionated CO_2_ laser in combination with topical and injected poly-l-lactic acid (PLLA) in a 44-year-old woman [53]. In this case, four sessions of both superficial and deep fractional CO_2_ laser treatment every three months with subsequent applications of a filler also resulted in an excellent patient satisfaction and significant cosmetic improvement persisted at follow-up three years after the final procedure without any adverse effects, and without the need for re-treatment [53].

**Figure 2:**
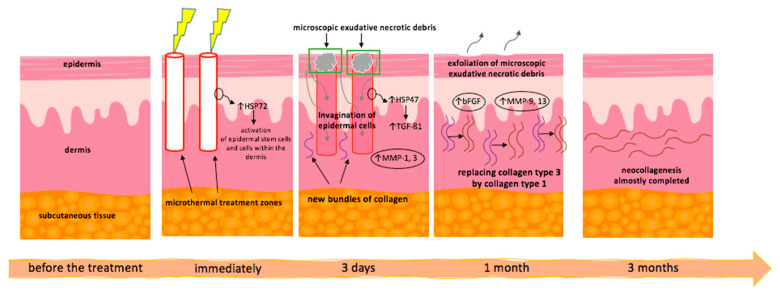
The mode of action of fractional lasers [32,44,47].

Recently, Shalaby et al. compared the efficacy of the fractional CO_2_ laser treatment (3 sessions) versus low-dose (30 J/cm^2^) UVA-1 therapy (30 exposures) in 17 patients with plaque or linear morphea based on the clinical and histopathological assessment [54]. The laser therapy led to significantly better clinical, histopathological, immunohistochemical, and ultrasound improvement. Comparing the two groups, there was a significant improvement in the clinical scores, regarding thickness, dermal atrophy, dyschromia, and erythema (based on the scores adopted and modified from LoSCAT), collagen homogenization, and patient satisfaction with a fractional CO_2_ laser that was superior to that of low-dose UVA-1 phototherapy. A very good response, i.e., ≥60% LoSCAT improvement, was obtained in 41% of morphea patients on the laser regimen versus 0% in the UVA-1 group, which showed a good response at best. Of note, according to histological variables, the fractional CO_2_ laser group exhibited statistically better improvement of collagen homogenization, whereas a decrease in inflammatory infiltrates was higher in the UVA-1-treated patients. Both fractional CO_2_ and UVA-1-treated lesions showed a significant but similar effect on a decrease in the mean profibrotic marker TGF-ß1 staining and increased MMP1 staining after treatment; however, the fractional CO_2_ laser group showed a statistically better decrease in ultrasonographically-assessed dermal thickness than the UVA-1 group. Additionally, patients in the CO_2_ laser group reported significantly higher satisfaction scores and a much lower incidence of post-inflammatory hyperpigmentation. However, compared to UVA1-treated individuals, they were more likely to experience pain during the treatment and itching within the first 24 h after the treatment [54]. Nonetheless, the advantage of fractional CO_2_ laser treatment over UVA-1 phototherapy may be concluded from this study. This may result from the more diverse action of fractional CO_2_ lasers on collagen remodeling, including: (i) an immediate mechanical effect due to formation of microthermal treatment zones (MTZ), which remove homogenized and fibrotic tissue, releasing the skin tightness, (ii) rise of multiple MMPs such as 1, 3, 9, and 13, with subsequent degradation of the thickened bundles of collagen as demonstrated by molecular studies [41,55], (iii) delayed, long-term effect on dermal collagen remodeling as a result of dynamic, controlled wound healing process via collagen production [51,56]. The latter seems to be associated with a gradual drop of TGF-ß1 within 1-month post-treatment after its initial rise in the first 3 days after laser procedure [35,41]. Moreover, the heat shock proteins (HSPs), such as HSP72 and HSP47, have been shown to be up-regulated around the MTZs in response to laser-induced coagulation, and are involved in both initiating as well as long-term collagen dermal remodeling [57,58]. The presumable mode of action of fractional lasers is summarized in Figure 2.

Laser-induced coagulation creates microthermal treatment zones (MTZs) in the skin. In response to damage, there is an increase in heat shock proteins (HSPs), firstly HSP72, which participates in the activation of epidermal stem cells and cells within the dermis, promoting rapid repair and collagen remodeling (its highest expression is about 3–14 days after the laser application). During the first day, special structures are formed, i.e., microepidermal necrotic debris (MEND), containing coagulated tissue. HSP47 expression is upregulated 4–7 days after laser treatment and is maintained for 3 months, resulting in increased accumulation of procollagen and collagen I and III. An initial increase of TGF-ß1 is seen in the first 3 days after treatment, with a gradual decline thereafter over a period of 30 days. Concentrations of MMP1 and MMP3, collagen-degrading enzymes, gradually increase over 3 days, peak on day 7, and decrease during the second week after laser. Levels of MMP 9 and MMP13 remain elevated for longer periods of time, ensuring the degradation of residual collagen. The increase in bFGF, which provides the coordination of initial migration and proliferation of endothelial cells, occurs later in time. Within a month, MEND exfoliation is entirely complete, and type 3 collagen is replaced by type 1. Complete replacement of the MTZ by fresh collagen fibers occurs after approximately 3 months.

There are also single case reports on the use of fractional Er:YAG laser in LoS. Kozarev presented a case of two women with morphea lesions on their lower legs who were treated with Er:YAG fractional lasers [59]. During the treatment they experienced a burning sensation but not intense pain. After interventions, erythema was observed, lasting from 2 to 10 days (mean 4.6 days), with intensity proportional to the number of laser beam passes. Based on the clinical and dermoscopic picture, a complete remission of lesions and improvement of skin structure were observed. The only adverse effect was transient hyperpigmentation [59].

The fractional Er:YAG laser laser was also effective in the treatment of Parry–Romberg syndrome, a rare variant of LoS of the head. Ghorbel et al. performed four sessions every two months with the use of 2940-nm Er:YAG laser on a 50-year-old woman with stabilized skin lesions located on the left cheek. After each session, marked improvement of the texture, color, and pliability was noted. Significant cosmetic improvement was maintained for 2 years after the last treatment [60].

#### 3.1.4. Alexandrite Laser

Alexandrite laser is a pulsed laser system that can be short-pulsed (Q-switched) or long-pulsed. It is considered as particularly effective for the treatment of superficial pigmented lesions, but it is also beneficial in the removal of vascular lesions as well as unwanted hair or tattoos [61,62]. Arpey et al. used a Q-switched alexandrite laser (755 nm) in a 22-year-old man with extensive atrophoderma Pasini and Pierini, presenting as multiple hyperpigmented and atrophic lesions on the trunk. After three exposures, a clear clinical improvement was observed, as a 50% diminution in hyperpigmentation was obtained. No adverse reactions were reported during the treatment cycle [63].

#### 3.1.5. Neodymium-Doped Yttrium Aluminum Garnet (Nd:YAG) 1064 nm Laser

The non-ablative 1064 nm Nd:YAG laser constitutes one of the most widely used devices in dermatological management and some of the reasons for its use include unwanted hair, vascular lesions, benign pigmented lesions, basal cell carcinoma, rhytids, cellulite, onychomycosis [64,65,66,67]. It is believed to act by passing thermal energy to small vessels and tissues in the upper dermis, which in turn stimulates fibroblasts in the dermis to promote collagen and elastin regeneration [64]. A particular advantage of Nd:YAG lasers is their safety for dark-skinned individuals, as the wavelengths of the infrared area are weakly attracted to melanin [68].

However, there is only one report of the Nd:YAG laser use in LoS. Bimbi et al. described a case of a 22-year-old female with a linear morphea lesion with apparent induration and macular hyperpigmentation located on her right lower limb that was unresponsive to topical therapy [69]. Satisfactory results were obtained following 5 sessions with long-pulsed Nd:YAG laser and pulsed light on the area of hyperpigmentation [69].

The studies included in the review are presented in Table 1.

## 4. Discussion

Nowadays, the use of laser devices for the treatment of cosmetic and non-cosmetic skin lesions is increasing, due to the minimally invasive character of treatment reflected in a lower risk of side effects, rapid healing of the targeted skin, and shortened recovery time [70,71,72]. The wide selection of laser devices and the possibility of matching the appropriate mode of action to the clinically observed skin changes is a great advantage [73]. This is particularly essential for LoS, a disease with many clinical faces and an incompletely understood etiology.

**Figure 3:**
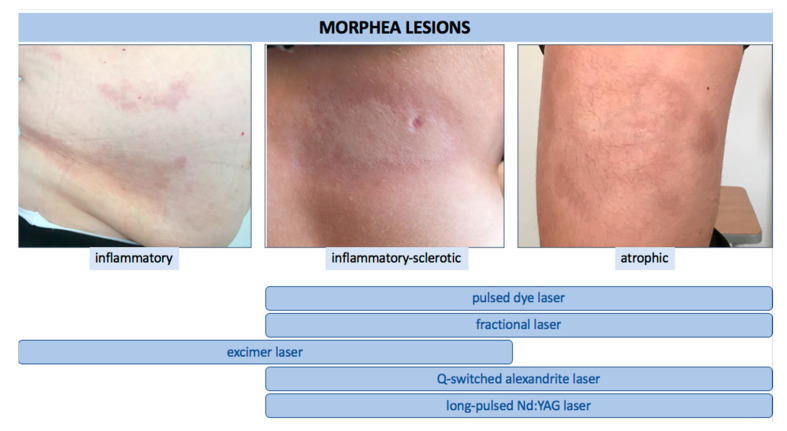
The use of various types of lasers according to clinical stage of LoS.

In this systematic review of the literature, we identified a total of twenty relevant studies using laser therapy for LoS. Eight studies were focused on the use of PDL, six on fractional lasers (CO_2_ and Er:YAG), four on excimer, and two on either alexandrite or Nd:YAG. The majority of these studies were single case reports or case series. Two studies were investigational [21,31] and one was of observational type [34]. We found only one comparative randomized controlled trial (fractional CO_2_ laser vs. UVA-1 therapy) [54]. In all reports, the authors emphasized the efficacy and high safety profile of the applied treatment. It is noteworthy that the therapy was also applied with good response and tolerance in pediatric patients [21]. Undoubtedly, significant variation in study design is evident, including indications, settings of laser parameters, the number of treatments, and the time intervals between them. Moreover, in some studies, laser therapy was used as an additional method to systemic or topical treatment. Therefore, the absolute effect of the laser application in LoS patients could not be assessed. Nonetheless, based on available literature reviewed in this article, and taking into account the mode of action of particular laser devices, we believe that: (i) for active, short-lasting, inflammatory LoS lesions (erythematous plaques), an excimer laser may be suggested, (ii) for active, short-lasting but sclerotic lesions, the use of excimer laser or PDL may be better considered, and on the other hand, (iii) inactive sclerotic lesions as well as hyperpigmented and atrophic ones, can be treated with PDL, alexandrite, Nd:YAG, or fractional lasers (Figure 3). The latter seems to be a particularly valuable therapeutic option in the case of limb contractures, which are severe complications of the disease, limiting patients’ mobility and reducing the quality of life. In addition, photomechanical fenestrations of the skin (vertical channels of ablation) created by fractional lasers can be used for laser-assisted drug delivery of topically applied formulation, such as PLLA, which can significantly sustain cosmetic improvement in morphea by synergistically increasing new collagen synthesis, resulting in better effectiveness of the applied treatment [54]. However, due to a lack of direct or head-to-head comparative studies, including the use of different types of lasers, case-control studies, or laser therapy against conventional methods (except the study of Shalaby et al. [54]) as well as the small number of patients included in reviewed studies, it is difficult to make clear recommendations. Up to now, there have also been no double-blind controlled trials that would allow validation of the reported effects.

There are several previous review articles considering the use of laser treatments in patients with LoS; however, none includes as many studies as ours. According to Zwischenberger et al., 585-nm long-pulse laser therapy is supported by level ≥3 of evidence, meaning there is insufficient evidence to support its efficacy and safety [74]. In the view of Creadore et al., the same level of evidence was obtained for CO_2_ fractional laser therapy for morphea-related contractures. On the other hand, level of evidence IB for CO_2_ laser treatment was specified for plaque, linear, and en coup de sabre types [11]. Furthermore, Rodríguez-Salgado et al. reported that, according to the Centre for Evidence-Based Medicine, Oxford, fractioned CO_2_ laser versus UVA1 is a therapy with the level of evidence 2B and the strength of recommendation B [75].

Based on this literature review, laser therapy may be a valuable treatment in some cases of clinically evident LoS lesions or those confirmed by histopathological examination, but it should be kept in mind that this treatment does not slow down the overall course of the disease and does not prevent the formation of new lesions. Therefore, in patients with aggressively progressive lesions, general treatment should be introduced, while laser therapy may be considered as an adjuvant method to treat cutaneous complications of LoS or for cosmetic reasons to provide a better quality of life for the patients. A short recovery time that allows an early return of patients to daily activities, and a single session that enables complete or at least partial remission sustained over a long period of time, certainly form the basis for better patient compliance compared to the systematic use of topical medications [76]. Moreover, in some cases, laser application enables the abandonment of systemic treatment, which may be associated with the occurrence of complications and often requires periodic check-ups. Thus, the effectiveness and safety of laser application should encourage the pursuit of controlled studies with a greater number of patients to better determine its role in the treatment of LoS.

## Figures and Tables

**Table 1:** Studies included in this review.

Study	Type of Study	No of the Patients Completing the Study	Patients Age [Years]	Morphea Type	Duration of Disease	Type of Laser Used	Parameters	No of Treatments	Results of Treatment	Adverse Effects	Additional Therapy
Eisen et al. [20]	Case report	1	41	Plaque	-	Pulsed dye laser (PDL)	wavelength 585 nm long-pulsed, fluence 5.0 J/cm^2^, spot size 10 mm, cryogen duration 30 ms	4	Softening and improvement in pigmentation of a lesion	-	-
Tawfik et al. [21]	Investigational	26	14–37	Plaque	1–5 years	Pulsed dye laser (PDL)	wavelength 585 nm, pulse duration 450 us, spot size 5 or 7, fluence 7.5–8.5 J/cm^2^	4–12, every 2 weeks	Improvement in clinical, histological, immunohistological aspects; high patients’ satisfaction scores	-	-
Kakimoto et al. [22]	Case report	1	6	Linear (En coup de sabre)	3 months	Pulsed dye laser (PDL)	wavelength 595 nm, spot 7 mm, fluence 8 J/cm^2^, pulse duration 1.5 ms, duration 40 us, delay 30 us	1	Alleviation of erythema	Blistering, hypopigmentation	-
Kim et al. [23]	Case report	1	24	Linear	4 months	Pulsed dye laser (PDL)	wavelength 595 nm, spot 7 mm, pulse width 10 msec, duration 30 ms, delay 30 ms	2	Minimal improvement; temporary alleviation of erythema	-	-
Pickert et al. [24]	Case report	1	2,5	Linear	6 months	Pulsed dye laser (PDL)	fluence 8.5 J/cm^2^, (I treatment), 9.0 J/cm^2^ (II treatment), 9.25 J/cm^2^ (III treatment), spot size 10 mm, pulse duration 1.5 ms	3	Alleviation of erythema	-	-
Nijhawan et al. [25]	Case report	1	6	Linear	7 months	Pulsed dye laser (PDL)	wavelength 595 nm, spot 7 mm, fluence 8.5 J/cm^2^, pulse duration 1.5 ms total 53 pulses	1	Subsequent resolution of the erythema	-	-
Ng et al. [26]	Case report	1	7	Linear	7 months	Pulsed dye laser (PDL)	-	3	Temporary alleviation of erythema	-	-
Miura et al. [27]	Case report	1	11	Linear	2 years	Pulsed dye laser (PDL)	wavelength 595 nm, spot 7 mm, fluence 5.5 J/cm^2^	2	Temporary alleviation of erythema; lesions became white and slightly shiny	-	-
Nisticò et al. [31]	Investigational	5	46	Plaque	-	Excimer	mean MED 0.3 J/cm^2^, mean starting dose 0.25 J/cm^2^, mean dose per session 1.5 J/cm^2^, mean total dose 10 J/cm^2^	8–12 (mean 7)	Four months after treatments, partial remission seen in 60% patients, whereas slight improvement in 40% patients	Residual hyperpigmentation	-
Hanson et al. [32]	Case report	1	17	Linear	4 years	Excimer	maximum 2200 mJ/treatment	In total 34; sessions twice weekly	Remission of active disease, a decrease in the size of the lesion and minimalizing of subjective symptoms	-	Methotrexate
Hajjar et al. [33]	Case report	1	28	Plaque	-	Excimer	300 mJ one treatment; then 260 mJ	16	Resolution of local inflammation	Erythema after the first treatment (300 mJ); after decreasing the dose to 260 mJ–none	Hydroxychloroquine 400 mg and calcipotriene/bethametasone ointment twice a day
Tatu et al. [34]	Observational	-	-	Plaque	-	Excimer	-	-	Improvement of inflammatory lesions	-	-
Kineston et al. [51]	Case report	1	27	Mixed	1 year	Fractional carbon dioxide	single pass, single pulse, no overlap, fluence 50-mJ, 5% density	1	Improvement in the range of motions, alleviating pain	-	Methotrexate, topical agents, UV-A1, physical therapy
Farmer et al. [52]	Case report	2	(1) 50 (2) 22	1) Linear 2) Linear	- -	both patients: fractional carbon dioxide	(1) pulse energy 15 mJ/cm^2^, density 15% (2) pulse energy 100 mJ/cm^2^, density 3%	(1) 1 (2) 5	(1) improvement in hyperpigmentation and asymmetry (2) significant improvement in hyperpigmentation, induration, and range of motions	-	(1) topical poly-l-lactic acid and injection of botulinum toxin (2) none
Yeager et al. [53]	Case report	1	44	Deep morphea	-	Fractional carbon dioxide	pulse energy 80 mJ, density 5%, pulse energy 50 mJ, density 10%	4	Significant cosmetic improvement	-	After the laser therapy, sudden application of topical and injected poly-l-lactic acid
Shalaby et al. [54]	A parallel intra-individual comparative randomized controlled trial	17	7–47	Plaque *n* = 12 Linear *n* = 3 En coup de sabre *n* = 2	6–96 months	Fractional carbon dioxide	power 25 W, dwelling time 500 msec, spacing 500 um	3	Significantly better clinical, histopathological, immunohistochemical results; high patients’ satisfaction scores	Pain during sessions (mild to moderate *n* = 17, marked *n* = 10), itch in first 24 h after laser treatment (*n* = 8), persistent erythema (*n* = 1), hyperpigmentation (*n* = 1)	-
Kozarev [59]	Case report	2	-	Plaque	-	Er:YAG fractional laser	scanning device in turbo 3 mode, pulse width 100 ms, fluence 24 J/cm^2^	3	complete remission	transient hyperpigmentation	-
Ghorbel et al. [60]	Case report	1	50	Parry Romberg Syndrome	5 years	Er:YAG fractional laser	wavelength 2940 nm, fluence 120 J/cm^2^, 12-mm diameter handpiece, coagulation level at 4	4	excellent cosmetic results- pigmentation normalization of the lesions; tightening, softening of lesions; texture improvement	Local; erythema lasting <1 week and pinpoint bleeding	-
Arpey et al. [63]	Case report	1	22	Atrophoderma Pasini-Pierini	8 years	Q-switched alexandrite laser	wavelength 755 nm, fluence 9.0 J/cm^2^, spot 3 mm, repetition rate 2 Hz	3	decrease the severity of hyperpigmentation by 50%	-	-
Bimbi et al. [69]	Case report	1	22	Linear	2 years	long-pulsed Nd:YAG associated with pulsed light	wavelength 1064 nm, fluence 14 J/cm^2^, spot 5 mm, pulse duration 0.8 ms.	5	treated skin more pliable, plaques softened, mobility restored and hyperpigmentation decreased (due to pulsed light)	-	-

## Data Availability

Data sharing not applicable.
